# O brave new world that has such machines in it

**DOI:** 10.1186/1756-0381-7-26

**Published:** 2014-11-17

**Authors:** James D Malley, Karen G Malley, Jason H Moore

**Affiliations:** Center for Information Technology, The National Institutes of Health, Bethesda, MD USA; Malley Research Programming, Inc., Rockville, MD USA; Departments of Genetics and Community and Family Medicine, Institute for Quantitative Biomedical Sciences, The Geisel School of Medicine, Dartmouth College, One Medical Center Dr., Lebanon, NH 03756 USA

Machine learning is a critical component of any biological data mining. Given its established advantages, there remain challenges that need to be addressed before it can be considered practical and persuasive.

Thus, not quite *The Tempest* (Act V, Scene One), above, but Shakespeare would still have understood: learning machines have achieved stunning success in a stunning range of areas, but they are still often—correctly—seen as strange and mysterious. They make predictions {tumor, not tumor} with ease and rapidity, but how do we understand the forecasts? How were the forecasts made, and how do they apply to this patient, with this set of symptoms and exposure factors? How does a low mean square error translate into guidance for patient treatment and care? How is any machine translated?

These questions point to an unfortunate separation between the advances of learning machines and the needs of biomedical research or patient care. We have written about the nearly invisible interest shown by computer science groups in the shared task of communication and joint application, and a disconnect between some statistical teaching practices and the needs of researchers and medical practitioners [1]. Statisticians and computer scientists need to move ahead—together—to provide methods for interpreting the results of analysis and computation. Thus, many good methods, such as random forests or penalized regression, do not transparently offer the subject-matter researcher with directly understood conclusions.

We suggest that solving this harder problem, the interpretation of models derived from learning machines and algorithms, is both fundamental and possible. We can, for example, move away from pure binary yes/no classification algorithms to probability machines [2]. These take the same data with zero/one outcomes [tumor, not tumor] and return consistent estimates of probability for the two events, doing so in a model-free context. From the same data, risk machines can then be deployed to estimate familiar endpoints as relative risk or log odds [3]. The point here is not promote any specific methods but to show that such methods do exist, that desaturate the obscurity of the black box machine and help return us to familiar terms and the language of inference.

All these arts, the simple and evolved, the practical and theory-driven, the computational and the analytic, need collaborative attention for interpretable biomedical research. Prospero, Miranda, Ariel, and perhaps even troubled and treacherous Caliban would have understood.
